# CBCT assessment of mandibular molar furcation following root canal retreatment using engine-driven instruments

**DOI:** 10.1590/1807-3107bor-2024.vol38.0087

**Published:** 2024-09-13

**Authors:** Paulo Otávio Carmo SOUZA, Mike Reis BUENO, Brunno Santos de Freitas SILVA, Luiz Eduardo GREGORIS, Nádia do Lago COSTA, Carlos ESTRELA

**Affiliations:** (a) Universidde Federal de Goiás – UFGO, Department of Stomatology Sciences, Goiânia, GO, Brazil .; (b) São Leopoldo Mandic University, Faculty of Dentistry, Department of Radiology, Campinas, SP, Brazil

**Keywords:** Root Canal Therapy, Root Canal Preparation, Cone-Beam Computed Tomography, Software

## Abstract

This study employed e-Vol DXS cone beam computed tomography (CBCT) software to assess dentin remnants in the furcation area of mesial canals in mandibular molars during root canal retreatment (RCR). Four groups (Reciproc®, ProTaper Next®, Race Evo®, Protaper Gold®) were subjected to RCR, and CBCT images were captured before (T1) and after (T2) treatment. Measurements of remaining dentin thickness at 1 mm and 3 mm below the furcation were scrutinized. Results revealed no significant differences in mean thicknesses of mesiobuccal (MB) and mesiolingual (ML) canals at 1 mm and 3 mm from the furcation pre-treatment (T1). Post-treatment (T2) showed analogous findings, with no significant differences in mean thicknesses. However, disparities were found between MB and ML canals at both distances, both before and after retreatment. In essence, the evaluated instruments exhibited safety in RCR, implying that they are appropriate for use in critical areas of mandibular molars without inducing excessive wear. This study underscores the reliability of these instruments in navigating danger zones during RCR, and contributes valuable insights for dental practitioners who handle complex root canal scenarios in mandibular molars.

## Introduction

The success of endodontic treatment depends on how well the microorganisms in the root canal system can be controlled.^1^ Achieving a good treatment outcome requires effective root canal preparation, combined with irrigating solutions and intracanal medication.^2^ The infectious process and the anatomical complexity in areas of difficult access to endodontic instruments pose challenges to sanitizing root canals.^1^

Many automated instruments have been manufactured with various NiTi alloys, some of which are heat treated, and/or are endowed with superelastic properties and a shape memory effect, or feature different kinematics designed to maintain the path of the root canal.^3^ Several instruments have been analyzed and tested, and the main innovations in their manufacture are based on surface heat treatments, nickel-titanium alloy microstructure, and design (more rhomboid sections and different shapes along the extent of the instrument, helical angles, and varying tapers).^4,5^

Their heat-treated surface is designed to add greater elasticity and greater cyclic and torsional fracture resistance to the instruments.^3^ Accordingly, the ProTaper Next® instrument is manufactured with a metal alloy using M-Wire technology^6^. It features both martensite and R-phase, with an eccentric, rectangular cross-section and regressive conicity, and is driven by continuous rotation.^6^ The Reciproc® instrument system is also made of a metal alloy using M-Wire technology, and is activated in reciprocating kinematics, with counterclockwise action.^3^

The evolution of nickel-titanium alloys promoted by the thermomechanical treatment process used in manufacturing the instruments alters the molecular structure of the alloy, and provides resistance to cyclic fatigue and greater flexibility, while reducing the shape memory effect, as exemplified by the Protaper Gold® instruments.^5^ RaceEvo® instruments are manufactured from nickel-titanium, and are heat-treated. They also receive electropolishing surface treatment, which improves cutting efficiency, and reduces manufacturing process defects, hence lowering apical transport.^7^ This instrument features heat treatment and a triangular cross-section with alternating cutting blades, and is driven in continuous rotation kinematics.^7^ It also has a special booster tip, which facilitates the progression of the instrument, and maintains the original curvature.^7^

Longitudinal and transverse shaping during root canal preparation aims to remove irregularities, flatten the root canal walls, and enhance the mechanical action on bacterial biofilm.^2^ The lateral limit of cervical widening must be appropriate, considering that the aspect viewed on the periapical radiograph does not represent an accurate reference of the real dentin thickness.^2^ Errors in operative procedures, and the failure of endodontic treatment, associated with clinical factors, have been discussed and categorized.^8^ The most common operative errors that should be highlighted are endodontic treatment planning and root canal preparation, in both the first intervention and the endodontic reintervention.^8^ Regarding root canal retreatment, the objective is to remove the filling material from the root canal, and reestablish the longitudinal and transverse shaping limit, aiming to control the microorganisms in a persistent infection.^8^

The term danger zone in mandibular molars refers to the zone in which the dentin thickness has a thinner amount of dentin in the distal wall of the mesial root of lower molars.^9^ This mandibular dentin thickness constitutes a risk factor for excessive wear, since there is a risk of root perforation if the area is enlarged excessively.^8^ Several methodologies have been used over the years to analyze the dentin remnants in danger zones of mandibular molars after using different instrumentation techniques.^9-21^

The incorporation of cone beam computed tomography (CBCT) into modern endodontics has had an unprecedented impact on endodontic planning, diagnosis, and treatment, by improving decision-making in complex clinical cases.^2-22^ Despite the technological advances in CBCT hardware, interpretation skills must be honed. Currently, CBCT interpretation is still influenced significantly by image visualization software. For example, even when a small field of view (FOV) is used in a state-of-the-art device, the original CBCT images can appear unclear because of the artifacts, thus requiring a series of adjustments to improve their quality.^22^

The e-Vol DXS CBCT software was developed with features that can improve image quality, such as adjustments for specific brightness and contrast, custom image thickness control, an image sharpening filter, and a noise reduction filter, among other resources.^22^ One of these filters is intended for measuring anatomical structures configured for micrometric units, and enables more effective planning in determining the longitudinal and transverse limits of the root canal preparation.^23^

The continuous search for a safe reference for root canal preparation involves obtaining information on anatomical aspects to avoid errors in operative procedures. The present study emphasizes that care should be taken to ensure the safety of new endodontic instruments, and make dentin wear safer in danger zones. It also addresses the application of CBCT software as a tool for determining and measuring the areas at risk for root perforation. The present study aimed to determine the dentin thickness remaining in the danger zone of mesial canals of mandibular molars after applying different instruments in root canal retreatment, by using a new CBCT software.

## Methods

The current investigation complied with the ethical guidelines set by the Helsinki Declaration. Approval for this study was obtained from the Research Ethics Committee at our institution, under reference number CAAE 46452621.2.0000.5083. The sample for this investigation comprised mandibular human molars (both first and second) extracted for several reasons. The teeth were sourced from the Emergency Service of the School of Dentistry at our university.

The CBCT scans of 84 mandibular molars were acquired after fixing the teeth on a 7-cm diameter double-wax layer platform. The inclusion criteria were mandibular molars with an intact pulp cavity, complete rhizogenesis, and mesial roots with a mild (R > 8 mm) or moderate radius of curvature (R > 4 mm and R ≤ 8 mm). The exclusion criteria were calcifications, teeth with a single-canal mesial root, internal or external root resorption, root fractures/cracks, incomplete rhizogenesis, endodontically treated teeth, and teeth with intraradicular posts.

DICOM format files were acquired using a PreXion 3D Elite 13-bit CT scanner (PreXion, San Mateo, USA). The tomograph was configured to acquire an image with an isotropic voxel of 0.146 mm, and an 81 mm high x 56 mm diameter FOV, during a 37-second exposure (at 512 exposures per acquisition), with a tube voltage of 90 kVp, 13 bits, current at 4 mA, focal point of 0.20 x 0, 20 mm, and total radiation beam filtration > 2.5 mm Al/eq. The images used the DICOM format and were post-processed using e-Vol DXS software (CDT Software, São José dos Campos, Brazil).

The teeth were opened and explored with a #15 manual file (Dentsply/Maillefer, Ballaigues, Switzerland). The glide path was performed using the WaveOne Gold Glider instrument (Dentsply/Maillefer, Ballaigues, Switzerland), and the root canal was prepared with Wave One Gold Primary (Dentsply/Maillefer, Ballaigues, Switzerland) applied by using the technique recommended by the manufacturer. The canals were flooded with a 2.5% sodium hypochlorite solution, and passive ultrasonic irrigation (PUI) was performed with an E1 ultrasonic tip (Helse Ultrasonic, Santa Rosa de Viterbo, Brazil) 3 times for 20 seconds each in each root canal, and subsequently filled with EDTA at 17% for 3 minutes (pH 7.2). The teeth were then filled with AH Plus® (Dentsply/Maillefer, Ballaigues, Switzerland) using the lateral condensation technique.

Root canal retreatment was conducted in all the groups 15 days following the initial root canal treatment, by using an R25 instrument (#25/variable taper) from the Reciproc® system (VDW, Munich, Germany). This procedure complied with the manufacturer’s instructions, including the specifications for speed and torque, and was performed in Reciproc mode ALL. This removal step of the filling material was performed based on the previous working length. After the instrument reached the working length in free rotation, the filling removal was concluded. Subsequently, the 84 teeth were distributed into 4 groups of 21 teeth each, and each group used another type of root canal re-preparation system (Table 1).

**Table 1:** Groups and repreparation systems used in root canal retreatment.

Group	Repreparation system
1 (n = 21)	Reciproc R25 + Reciproc R40 ® (VDW, Munich, Germany)
2 (n = 21)	Protaper Next ® (X2 X3 e X4) (Dentsply/Maillefer, Ballaigues, Switzerland)
3 (n = 21)	Race Evo (#25.04, #30.04, #40.04) ® (FKG Dentaire, La Chaux-de-Fonds, Switzerland)
4 (n = 21)	Protaper Gold ® (F2, F3 e F4) (Dentsply/Maillefer, Ballaigues, Switzerland)

**Figure 1:**
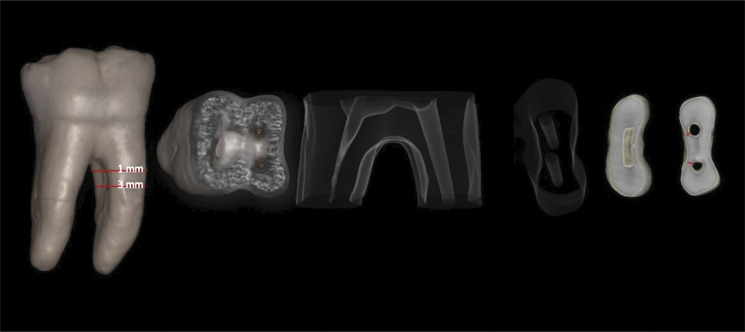
Illustration of a cone beam computed tomography image with 3D reconstructions of areas measured at 1 and 3 mm below the furcation: measurement of dentin thickness in CBCT images using the e-Vol DX software filter.

**Figure 2:**
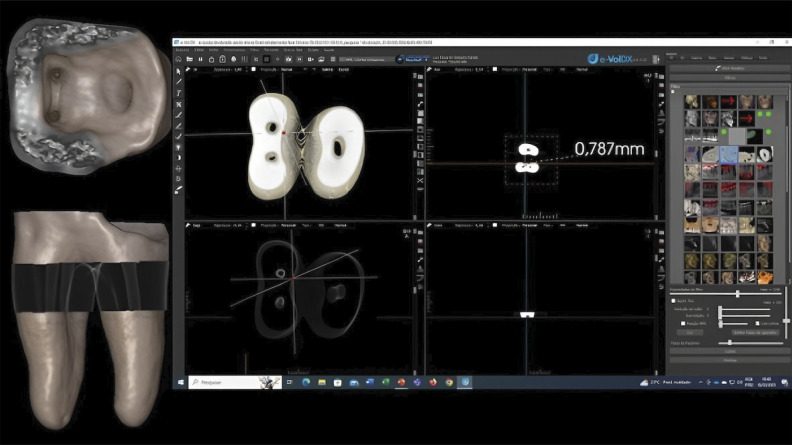
Illustration showing the synchronization of the 2D mode adjusted to have the same dimension as the 3D image. Dimensions were calibrated until the 3D and 2D modes matched.

**Figure 3:**
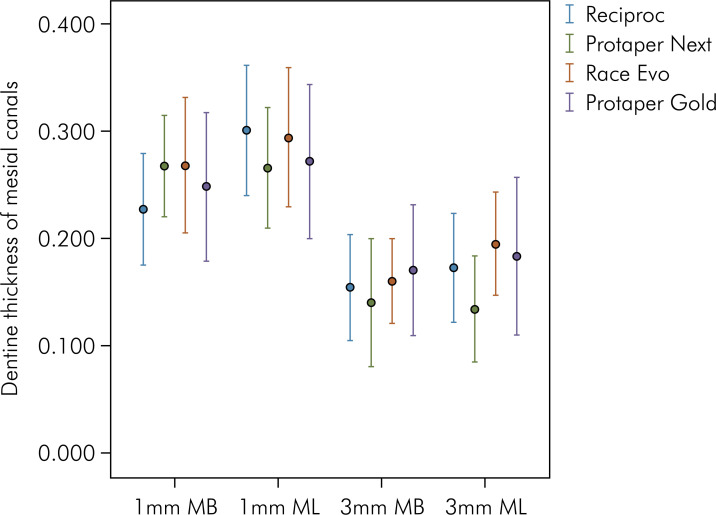
Representation of the values of the different dentin thicknesses (mm) before and after preparation of each group (Reciproc, Protaper Next, Race Evo and Protaper Gold), levels (1 and 3 mm from the furcation) or root canals (MB and ML).

After root canal retreatment, the CBCT scans were acquired in the same way used to determine the inclusion/exclusion criteria, described above. The measurements were obtained by aligning each sample with the three anatomical orientation planes (axial, coronal, and sagittal axis), and were standardized so that the long axis of the sample remained perpendicular to the ground, the mesial canals were aligned from the axial point of view, and the sagittal and coronal planes could be used to correct the parallax error. The dentin thickness on the distal wall of the mesial root of mandibular molars was measured on the CBCT images before and after root canal retreatment. The blooming artifact reduction (BAR) level 2 filter was used, and the chosen measurement region was 1 mm and 3 mm below the furcation, defined according to the three anatomical orientation planes and the 3D image. The diameter of the dentin thicknesses in the CBCT images was measured using the e-Vol DXS CBCT software filter,^22^ according to the method proposed by Bueno et al.^23^ After applying this methodology, linear measurements of the dentin thicknesses were obtained in the 4 groups at 1 mm and 3 mm below the furcation on the distal walls of the mesial root canals of the mandibular molars, at T1 and T2 (Figures 1, 2 and 3). All the imaging exams were analyzed by two experienced and previously calibrated observers (a radiologist and an endodontist).

The mean and the standard deviation values of the variables were obtained. Data normality was assessed using the Kolmogorov-Smirnov test. The difference between the independent groups was assessed using the Bonferroni one-way post hoc ANOVA test or the Kruskal-Wallis test. The difference between the dependent variables was evaluated using the t-test for paired samples or the Wilcoxon test. P values < 0.05 were considered significant. Statistical analysis of the data was performed using the Statistical Package for the Social Sciences software, version 20 (SPSS, Chicago, USA).

## Results

The results are shown in Tables 2, 3 and 4. Table 2 shows the mean dentin thicknesses before root canal preparation for each group, at each level, and in each root. A comparison among the groups showed no difference between the mean thicknesses of the mesiobuccal (MB) and mesiolingual (ML) canals, neither at 1 mm (p = 0.693 and p = 0.718), nor at 3 mm from the furcation (p = 0.594 and p = 0.408).

**Table 2:** Mean and standard deviation of dentin thickness (mm) before preparation of each group (Reciproc, Protaper Next, Race Evo, Protaper Gold), level (1 and 3 mm from the furcation), and root canal (MB and ML).

Variable	Reciproc	Protaper Next	Race Evo	Protaper Gold	p-value
	−*X* ± *S*	−*X* ± *S*	−*X* ± *S*	−*X* ± *S*	
1 mm					
MB	1.031 ± 0.165	0.984 ± 0.143	1.043 ± 0.232	1.037 ± 0.160	0.693*
ML	1.024 ± 0.225	0.988 ± 0.209	1.038 ± 0.219	1.019 ± 0.228	0.718**
3 mm					
MV	0.968 ± 0.197	0.897 ± 0.245	0.989 ± 0.308	0.972 ± 0.170	0.594*
MB	0.928 ± 0.232	0.866 ± 0.258	0.989 ± 0.278	0.923 ± 0.218	0.408**

−*X*: average. *S*: standard deviation. *ANOVA test. **Kruskal-Wallis test

**Table 3:** Mean and standard deviation of dentin thickness (mm) after preparation of each group (Reciproc, Protaper Next, Race Evo, Protaper Gold), level (1 and 3 mm from the furcation), and root canal (MB and ML).

Variable	Reciproc	Protaper Next	Race Evo	Protaper Gold	p-value
	−*X* ± *S*	−*X* ± *S*	−*X* ± *S*	−*X* ± *S*	
1 mm					
MB	0.804 ± 0.176	0.717 ± 0.167	0.775 ± 0.280	0.789 ± 0.161	0.518*
ML	0.723 ± 0.177	0.722 ± 0.224	0.743 ± 0.234	0.748 ± 0.205	0.969*
3 mm					
MB	0.814 ± 0.185	0.757 ± 0.201	0.829 ± 0.297	0.802 ± 0.153	0.724*
ML	0.756 ± 0.183	0.732 ± 0.212	0.795 ± 0.251	0.740 ± 0.182	0.651**

−*X*: average. *S*: standard deviation. *ANOVA test. **Kruskal-Wallis test

**Table 4:** Mean and standard deviation of subtraction of dentin thickness (mm) before and after retreatment of each group (Protaper Next, Race Evo, Reciproc, Protaper Gold), and level (1 and 3 mm from the furcation).

Variable	Reciproc	Protaper Next	Race Evo	Protaper Gold	p-value
	−*X* ± *S*	−*X* ± *S*	−*X* ± *S*	−*X* ± *S*	
1 mm					
MV	0.227 ± 0.114	0.267 ± 0.104	0.268 ± 0.139	0.248 ± 0.156	0.490**
ML	0.301 ± 0.134	0.266 ± 0.123	0.294 ± 0.143	0.272 ± 0.153	0.784**
3 mm					
MV	0.154 ± 0.108	0.140 ± 0.131	0.160 ± 0.088	0.170 ± 0.138	0.609**
ML	0.173 ± 0.112	0.134 ± 0.108	0.195 ± 0.106	0.183 ± 0.157	0.407*

Table 3 shows the mean thickness after root canal retreatment in each group, according to the thickness level and the root canal. A comparison among the groups shows no difference in the mean thicknesses of the MB and ML canals at 1 mm (p = 0.518 and p = 0.969) or 3 mm (p = 0.724 and p = 0.651) from the furcation. Moreover, there were no root perforations. Table 4 and Figure 3 show the mean and standard deviation of subtraction values of dentin thickness (mm) before and after root canal retreatment at 1 mm and 3 mm below the furcation, for each group, thus evidencing that there was no difference among the mean thickness values.

## Discussion

The nickel-titanium instruments (Reciproc®, Protaper Next®, Race Evo® and Protaper Gold®) showed similar performance in maintaining the average dentinal thickness at the end of the mesial canal retreatment of mandibular molars. The mean thicknesses measured revealed values exceeding 0.717 mm at distances of 1 mm and 3 mm from the furcation area. At 1 mm and 3 mm below the furcation, the average thickness after wear did not exceed 0.301 mm and 0.195 mm, respectively. Although the instruments studied displayed different kinematics, alloys, and designs, they did not show any difference in wear after root canal retreatment, levels (1 and 3 mm from the furcation), or root canals (MB and ML), thus demonstrating the safety of the instruments in the danger zone of mandibular molars.

The concern to carry out a safer and more controlled preparation in the danger zone of mandibular molars has led to proposing new anti-curvature instrumentation protocols of the root canal to avoid weakening and/or perforation in this anatomical region.^9^ Several studies compared instrumentation techniques to determine the best way of evaluating the dentin thickness at a level of 1 to 5 mm below the furcation.^10,11^ The methodologies applied to measure the remaining dentin in the danger zone in these previous studies showed that the areas most susceptible to perforations were found between 2 and 3 mm below the furcation region. Several methodologies have been described to evaluate endodontic instrumentation and its impact on the endodontic anatomy.^9,21^

With the advent of CBCT and advances in CT scanner resolutions, the ability to measure dimensional anatomical structures of the root canal has become increasingly more accurate.^23^ Thus, studies have evaluated the dentin thickness of the danger zone in the mesial roots of mandibular molars by using CBCT, different fields of view, and smaller voxels.^12-16,18,20^ The association of e-Vol DXS CBCT software with CBCT helps determine anatomical structures, since the software features certain controls to adjust brightness, achieve specific contrast, control thickness, reduce noise reduction, personalize image sharpness, and apply 3D rendering filters that can enhance the reliability of measuring structures in micrometric units, and be replicated in vivo studies.^20,23^ A CBCT scan can determine the apical anatomical diameter, and plan the lateral enlargement limit to select a preparation system that avoids excessive transverse wear, and is compatible with the root canal geometry and pathological conditions.^23^

Previous studies^9,21^ have analyzed levels between 1 and 5 mm from the furcation region on the distal wall of the mesial root of mandibular molars, and have observed that the mean thickness at 2 mm below the furcation is from 0.78 to 1.27 mm. Lim and Stock^11^ indicated that a minimum dentin thickness of 0.3 mm should be preserved after root canal preparation to resist condensation forces during obturation, and hence avoid perforation or vertical root fracture.^11^ At the end of the retreatment of mesial canals of mandibular molars with different preparation instruments, the thicknesses showed results greater than 0.717 mm at levels of 1 and 3 mm from the furcation area.

The initial thickness of the danger zone of mesial roots of mandibular first molars was evaluated with Chinese patients by using a CBCT.^18^ The results showed that there were no differences between the MB and ML canals, and that the thinnest thicknesses were at a level of 3 to 4 mm below the furcation.^18^ The dentin thickness of the danger zone was evaluated using another CBCT scan, and the results showed less dentin thickness in the danger zone located at a level of 3 mm below the furcation, with a mean value of 0.81 mm.^18^ The results of the present study were similar to those of Zhou et al.,^18^ considering that the MB and ML canals also showed no difference. The mean dentin thickness (0.941 mm ± 0.240) at a level of 3 mm was lower than at the level of 1 mm below the furcation (1.020 mm ± 0.191). The danger zone of lower molars was also studied using microcomputed tomography (micro-CT), and the results showed dentinal thicknesses in the MB canals that ranged from 0.67 to 1.93 mm, with a mean of 1.13 ± 0.21mm, and in the ML canals, from 0.77 to 1.89 mm, with a mean of 1.10 ± 0.21 mm, mainly in the middle third of the root (4.37 ± 1.68 mm below the furcation).^17^ In this study, both the mesial and the distal walls of the mesial root were evaluated, and the measurements were taken at a level of 1 to 7 mm below the furcation.

Several studies have performed measurements of dentin thicknesses using CBCT images with Gates-Glidden drills, Largo burs and NiTi instruments to prepare the cervical third.^12,13,15^ The results have stressed that transversal shaping in the danger zone must be limited, and that the instrument taper must be selected correctly to reduce the occurrence of lateral perforation of this thin zone. WaveOne® instruments were used in continuous rotation and reciprocating kinematics to prepare the mesial canals of lower molars. Subsequently, the wear caused to the distal wall at a level of 2 and 4 mm below the furcation was evaluated using CBCT. The results showed that the alteration of the kinematics did not promote any statistical difference in the remaining dentin after preparation at a level of 2 mm below the furcation. With reciprocating kinematics, WaveOne wore 0.26 ± 0.14 mm, and in continuous rotation, 0.28 ± 0 .13 mm.^14^ These results are in line with those of the present study, since there were no differences in the types of driving systems (continuous rotation, Protaper Next®, Race Evo®, Protaper Gold®, or reciprocating kinematics, Reciproc®). Silva et al.^19^ evaluated the systems using a micro-CT to compare the influence of the design of the cavity preparation on the remaining dentin thickness after root canal preparation with Reciproc Blue® R25 and R4019. The results showed that the shape of the crown opening does not influence the wear capacity of endodontic instruments in the danger zone of lower molars. In addition, even when using heat-treated endodontic instruments, the remaining thicknesses showed results between 0.5 to 1 mm, similar to studies using instruments without heat treatment (Gates-Glidden and Largo).^12,13,15^

A previous study^20^ using e-Vol DXS software to analyze thickness measurements, also evaluated the wear behavior of the following instruments after root canal preparation: ProTaper Next®, Reciproc Blue®, Bio-Race®, and WaveOne Gold®^20^. The results showed that the remaining dentin thickness in the prepared canals was greater than 0.670 mm in all the groups. There was a greater amount of dentin removed at 1 mm below the furcation (0.734 ± 0.191), even in relation to the thinnest dentin thickness at 3 mm from the furcation after preparation (0.715 ± 0.186).^20^ The results showed that the initial thickness averaged 0.900 mm ± 0.191 at the level of 3 mm below the furcation, and 1.035 mm ± 0.184 at the level of 1 mm below the furcation. Although the present study evaluated teeth after root canal retreatment, the results corroborate those described by Sousa et al.,^20^ that is, the initial thickness presented an average of 0.941 ± 0.240 mm at a level of 3 mm below the furcation, and an average of 1.020 ± 0.191 mm at a level of 1 mm below the furcation. This finding shows that the area at a level of 3 mm below the thinnest furcation has a greater risk of lateral perforation during the root canal enlargement. Furthermore, the different types of root canal preparation and re-preparation systems did not show any differences in the remaining dentin thickness, thus confirming the safety of the endodontic instruments used.

Although several studies report on determining the thickness of the danger zone in lower molars, specific criteria are standardized differently, including the type of tomograph and software to be used, the measurement method, and the instrumentation systems. The application of modern technological resources, such as the new CBCT software, enables a precise clinical analysis of these anatomical regions, which must be treated carefully and must retain an anatomically safe thickness. The present study offers a preliminary method for determining the thickness of the danger zone in teeth that have undergone root canal retreatment. It was based on a standard model for using e-Vol DXS software, and describes the method as a previously performed dynamic navigation along the root canals, by using measurements of each sample.^20,22,23^ One limitation of this study was the inability to conduct in situ measurements on the teeth before and after the procedure in a non-destructive manner. This constraint prevented a direct comparison between CBCT measurements and the gold standard.

The clinical application of the current assay findings stresses that the danger zone preparation should be performed with great care to avoid excessive preparation in cases of root canal retreatment. The best clinical therapeutic option is one that prevents failure in achieving the best operative procedure outcome.

## Conclusions

The transverse enlargement performed in the root canal retreatment of mandibular molars did not cause excessive wear in the danger zone of the mesial root and maintained an average thickness above 0.717 mm at 1 and 3 mm from the furcation area. The instruments tested showed similar behavior for wear after root canal retreatment, thus making them safe for use in danger zones of mandibular molars. Post-processing CBCT software has enabled precise determination of dentin thickness measurements and validation of the safety of the tested instruments.

## References

[1] Peters OA (2004). Current challenges and concepts in the preparation of root canal systems: a review. J Endod.

[2] Estrela C, Holland R, Estrela CR, Alencar AH, Sousa-Neto MD, Pécora JD (2014). Characterization of successful root canal treatment. Braz Dent J.

[3] Gavini G, Santos MD, Caldeira CL, Machado ME, Freire LG, Iglecias EF (2018). Nickel-titanium instruments in endodontics: a concise review of the state of the art. Braz Oral Res.

[4] Çapar ID, Arslan H (2016). A review of instrumentation kinematics of engine-driven nickel-titanium instruments. Int Endod J.

[5] Zupanc J, Vahdat-Pajouh N, Schäfer E (2018). New thermomechanically treated NiTi alloys - a review. Int Endod J.

[6] Elnaghy AM, Elsaka SE (2014). Evaluation of root canal transportation, centering ratio, and remaining dentin thickness associated with ProTaper Next instruments with and without glide path. J Endod.

[7] Basturk FB, Özyürek T, Uslu G, Gündogar M (2022). Mechanical properties of the new generation RACE EVO and R-motion nickel-titanium instruments. Materials (Basel).

[8] Dioguardi M, Stellacci C, La Femina L, Spirito F, Sovereto D, Laneve E (2022). Comparison of endodontic failures between nonsurgical retreatment and endodontic surgery: systematic review and meta-analysis with trial sequential analysis. Medicina (Kaunas).

[9] Abou-Rass M, Frank AL, Glick DH (1980). The anticurvature filing method to prepare the curved root canal. J Am Dent Assoc.

[10] Kessler JR, Peters DD, Lorton L (1983). Comparison of the relative risk of molar root perforations using various endodontic instrumentation techniques. J Endod.

[11] Lim SS, Stock CJ (1987). The risk of perforation in the curved canal: anticurvature filing compared with the stepback technique. Int Endod J.

[12] Sanfelice CM, Costa FB, Só MVR, Vier-Pelisser F, Bier CAS, Grecca FS (2010). Effects of four instruments on coronal pre-enlargement by using cone beam computed tomography. J Endod.

[13] Flores CB, Montagner F, Gomes BP, Dotto GN, Schmitz MS (2014). Comparative assessment of the effects of Gates-Glidden, Largo, LA-Axxess, and New Brazilian Drill CPdrill on coronal pre-enlargement: cone-beam computed tomographic analysis. J Endod.

[14] Shantiaee Y, Dianat O, Paymanpour P, Nahvi G, Ketabi MA, Kolahi Ahari G (2015). Alterations of the danger zone after preparation of curved root canals using WaveOne with reverse rotation or reciprocation movements. Iran Endod J.

[15] Akhlaghi NM, Bajgiran LM, Naghdi A, Behrooz E, Khalilak Z (2015). The minimum residual root thickness after using ProTaper, RaCe and Gates-Glidden drills: a cone beam computerized tomography study. Eur J Dent.

[16] Pinto SSL, Lins RX, Marceliano-Alves MFV, Guimarães MD, Fonseca BA, Radetic AE (2018). The internal anatomy of danger zone of mandibular molars: A cone-beam computed tomography study. J Conserv Dent.

[17] De-Deus G, Rodrigues EA, Belladonna FG, Simões-Carvalho M, Cavalcante DM, Oliveira DS (2019). Anatomical danger zone reconsidered: a micro-CT study on dentine thickness in mandibular molars. Int Endod J.

[18] Zhou G, Leng D, Li M, Zhou Y, Zhang C, Sun C (2020). Root dentine thickness of danger zone in mesial roots of mandibular first molars. BMC Oral Health.

[19] Silva EJ, Lima CO, Barbosa AF, Lopes RT, Sassone LM, Versiani MA (2022). The Impact of TruNatomy and ProTaper Gold Instruments on the Preservation of the Periradicular Dentin and on the Enlargement of the Apical Canal of Mandibular Molars. J Endod.

[20] Sousa VC, Alencar AHG, Bueno MR, Decurcio DA, Estrela CRA, Estrela C (2022). Evaluation in the danger zone of mandibular molars after root canal preparation using novel CBCT software. Braz Oral Res.

[21] Silva EJ, Lima CO, Barbosa AF, Moreira T, Souza EM, De-Deus G (2022). Influence of access cavity preparation on the dentine thickness of mesial canals of mandibular molars prepared with reciprocating instruments. Int Endod J.

[22] Bueno MR, Estrela C, Azevedo BC, Diogenes A (2018). Development of a new cone-beam computed tomography software for endodontic diagnosis. Braz Dent J.

[23] Bueno MR, Estrela CR, Granjeiro JM, Sousa-Neto MD, Estrela C (2019). Method to Determine the root canal anatomic dimension by using a new cone-beam computed tomography software. Braz Dent J.

